# A Reconstruction Method for the Estimation of Temperatures of Multiple Sources Applied for Nanoparticle-Mediated Hyperthermia

**DOI:** 10.3390/molecules23030670

**Published:** 2018-03-16

**Authors:** Idan Steinberg, Gil Tamir, Israel Gannot

**Affiliations:** 1 Multimodality Molecular Imaging Lab (MMIL), Department of Radiology, School of Medicine, Stanford University, Stanford, CA 94305-5427, USA; idanstei@stanford.edu; 2 The Laboratory for Optics and Lasers in Medicine , Department of Biomedical Engineering, Tel Aviv University, Tel-Aviv 6997801, Israel; gil.tammir@gmail.com; 3 Department of Electrical and Computer Engineering, Whiting School of Engineering, Johns Hopkins University, Baltimore, MD 21218-2608, USA

**Keywords:** thermal imaging, frequency modulation, reconstruction, nanoparticles

## Abstract

Solid malignant tumors are one of the leading causes of death worldwide. Many times complete removal is not possible and alternative methods such as focused hyperthermia are used. Precise control of the hyperthermia process is imperative for the successful application of such treatment. To that end, this research presents a fast method that enables the estimation of deep tissue heat distribution by capturing and processing the transient temperature at the boundary based on a bio-heat transfer model. The theoretical model is rigorously developed and thoroughly validated by a series of experiments. A 10-fold improvement is demonstrated in resolution and visibility on tissue mimicking phantoms. The inverse problem is demonstrated as well with a successful application of the model for imaging deep-tissue embedded heat sources. Thereby, allowing the physician then ability to dynamically evaluate the hyperthermia treatment efficiency in real time.

## 1. Introduction

Cancer is one of the leading causes of morbidity and mortality worldwide [1]. Today’s clinicians are armed with multiple treatment options to alleviate the burden of solid malignant tumors. Despite the ever-growing number of available techniques, the most effective treatment is still a complete resection of the malignant mass with negative borders [2]. Unfortunately, many times complete removal is impossible, especially for diffused tumors or sensitive anatomic locations. Thus, other methods are used to supplement resection before or after the procedure. Most common are locally applied radiation therapy [3,4] or systemic chemotherapy [5,6].

A less common method which is of potential interest, is focused hyperthermia [7,8]. Such a method takes a much lower toll on the patient’s body and thus greatly improves his life quality while reducing the burden on the healthcare system. Hyperthermia takes advantage of the fact that the sensitivity of tumors to temperature elevation is higher than that of healthy tissue [9], i.e. in the temperature range of 42–47 °C a cancerous tissue will experience reduced viability while healthy tissue is expected to only experience reversible damage [10]. A focused and localized hyperthermia at the tumor sites makes it much more effective. Thus, in recent years, targeted hyperthermia approaches have been developed. Specifically, Nanoparticle Mediated Hyperthermia (NMH) uses targeted Super Paramagnetic Iron Oxide Nanoparticles (SPIONs) excited by external Radio Frequency (RF) fields to focus the generated heat at the tumor sites [11,12,13]. SPIONs are the only type of nanoparticles which currently have the necessary regulatory approvals for human use. This is due to their small size which allows renal clearance and non-toxic surface properties. Other focused approaches include photodynamic therapy (PDT) or brachytherapy. PDT uses light-sensitive drugs which are applied topically or systemically and when concentrating in tumor areas are activated by external light and release singlet oxygen [14,15,16]. PDT is often restricted to shallow tumors due to the extreme attenuation of light inside the tissue. Brachytherapy and other similar methods use miniaturized radiation sources (often called “seeds”) embedded inside the organ [17,18]. Such methods are much more invasive.

Previously, using a thermal camera and inverse modeling we were able to image NMH both in tissue-like phantoms and in vitro models [19]. We were able to localize these particles through the analysis of features of the temperature images [20,21,22]. We have shown that NMH can reduce tumor volume and aggressiveness [13].

In practice, all the above methods are being used without any real-time feedback system that may indicate whether the tumor is responding and the treatment is successful. The response is usually evaluated through imaging or biopsy after the whole set of the planned treatment has ended. Specifically, for hyperthermia, the temperature at the tumor vicinity is unknown as well as unconfined to the tumor region and thus, damage may occur to the surrounding tissue, or temperature might be insufficient for treatment. This work aims to fill this void. Specifically, we seek to increase the efficiency of NMH through control of the temperature at one or more tumor foci based on real-time estimation of the temperature in the tumor vicinity through the thermal images.

Previous work [19] proved the ability to predict the source’s depth alone in a single spherical heat source scenario, by means of classification of temperature rise time curve to a supervised training set. Such method relies on a large, diverse dataset of training examples (temperature measurements acquired from tissue-like phantom/in vitro experiments or simulations) which limits its generality and ability to handle more abstract scenarios (distributed, non-uniform heat source). We have also proposed using acoustical signal detection through a set of hydrophones around the organ of interest [23]. Our proposed solution, based on an analytic physical model, may solve the mentioned problem as it accounts for the abstract set of variables parameterizing the heat transfer equation. A time-harmonic model, i.e., heat source power which is a time-dependent sinusoidal function, was formulated in order to analyze temperature measurements in a quasi-steady state. Time-harmonic heat stimulation offers a high Signal to Noise Ratio (SNR) by means of Fourier transforming the measurements from time to frequency domain. The signal’s energy concentrates in a narrowband fashion and therefore can be well above from the noise floor. In addition, time-harmonic formulation simplifies the heat transfer solution, which enables the addition of complexity to the model (multilayer heat transfer).

The rest of the manuscript is arranged as follows. First, we present a rigorous derivation of the fast time-harmonic analytical model. Then the experimental setup and signal processing scheme are described. Finally, we experimentally assess the method’s ability to separate thermal sources in the lateral and axial directions. Tests are performed both in silico and on tissue-mimicking phantoms. Finally, the estimated temperatures of each of separate tumor foci are tested as well to demonstrate the ability to provide accurate feedback in real-time.

## 2. Materials and Methods

### 2.1. Time-Harmonic Analytical Model

**Figure 1:**
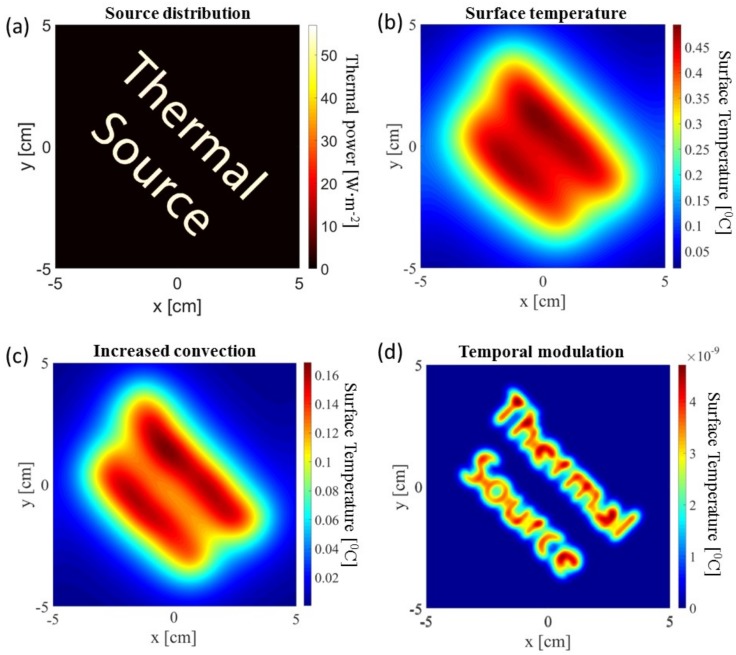
Temperature maps of simulated heat source embedded 8 mm below the surface. (**a**) The thermal source distribution over a 10 × 10 cm area; (**b**) The resulting surface temperature increase; (**c**) The same increase in temperature after a four-fold increase in the heat convention; (**d**) The (temporal) Fourier coefficient of the surface temperature at 1/3 Hz resulting from a source modulation at the same frequency.

Estimating the three-dimensional temperature distribution based on the surface temperature measured by a thermal camera is non-trivial. This is due to two major reasons: (A) the surface temperature is very much affected by external environmental conditions which might be unrelated to the internal temperature distribution that we seek to estimate and (B) the temperature is known to follow the heat equation which acts as a narrow low-pass spatial filter. Thus, even in the absence of external environmental perturbations—the two-dimensional surface temperature distribution is heavily blurred and axially integrated version of the three-dimensional one. The high-resolution temperature distribution that we seek. Figure 1 exemplifies these effects.

We make an important observation to overcome these effects. While the spatial distribution of the sources is unknown; the temporal distribution is known and externally controllable. Thus, by modulating the excitation RF field, one can take advantage of a known perturbation and filter out the signal components (which are linearly dependent on the modulation) from the noise component which are independent of the modulation. A straightforward application of such a technique utilizes a narrowband excitation waveform followed by filtering out all other frequencies in the measurement. This allows coherent increase of the signal to noise ratio (SNR) or resolution on the expanse of measurement time. This is, in contrast to simple non-coherent averaging of frames which will provide a square root increase in SNR and won’t improve resolution. 

Time-harmonic thermal stimulation leads to thermal diffused waves [24,25]. It allows us to apply the wave approach. Diffused waves are damped waves with a complex wavenumber. By increasing the frequency, one can increase the real part of the wavenumber (or decrease the wavelength) and thus improve resolution. However, since the imaginary part (which is responsible for the damping) is increasing with frequency as well, the damping will grow and additional images will be needed to compensate for the reduction in SNR.

**Figure 2:**
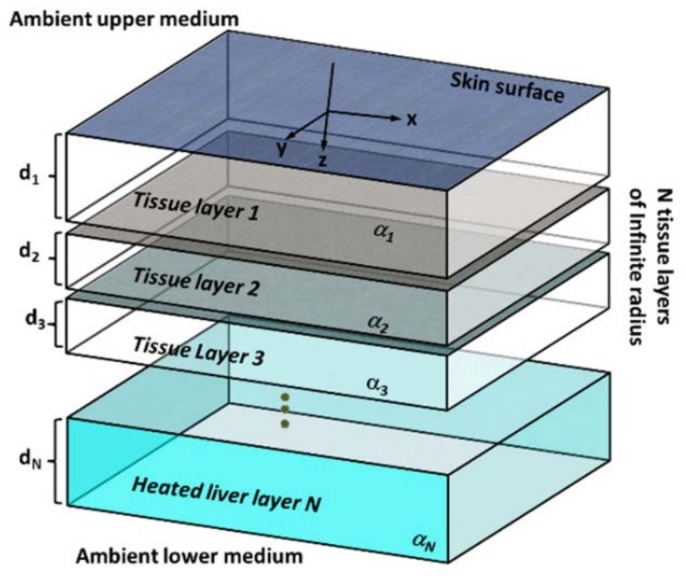
Analytical multi-layered thermal diffusion model geometry.

We’ve developed an analytic multilayered model. Such a model is applicable to many anatomic locations and a wide range of other applications. Since the model is based on a closed-form analytical expression it can calculate the heat source and/or temperature distributions at various depths in a rapid manner and thus can potentially allow a thermographic tomography of small regions. The model geometry is described in Figure 2.

In order to derive the analytical multi-layered thermal diffusion model, let us first consider Penne’s bio-heat equation:

(1) $$\begin{array}{c} \underset{\begin{array}{l} \text{change in}\\ \mathrm{temperature} \end{array}}{\underset{︸}{\rho_{T}C_{T}\frac{\partial }{\partial t}\tau_{T}\left(r,t\right)}}-\underset{\text{conduction to tissue}}{\underset{︸}{\kappa_{T}\nabla^{2}\tau_{T}\left(r,t\right)}}-\underset{\text{convection by perfusion}}{\underset{︸}{f_{B}\rho_{B}C_{B}\left[\tau_{B}\left(r,t\right)-\tau_{T}\left(r,t\right)\right]}}\\ =\underset{\text{metabolic heat sources}}{\underset{︸}{q_{MET}\left(r,t\right)}}+\underset{\text{extrnal heat sources}}{\underset{︸}{q_{EXT}\left(r,t\right)}} \end{array}$$

This equation describes the temperature distribution inside the tissue τT(r,t)[K0] in time and space. The subscript T is related to the tissue properties–thermal conductivity: κT[W⋅m−1⋅K−10], density: $\begin{array}{cc} \rho_{T} & \left[kg\cdot m^{-3}\right] \end{array}$ and heat capacitance: CT[J⋅kg−1⋅K−10]). The subscript b is related to arterial blood rather than the tissue—$\begin{array}{cc} f_{B} & \left[Hz\right] \end{array}$ blood perfusion rate. Temperature changes are due to heat conduction to/from neighboring tissues, heat convection by arterial blood and due to a heat source, such as metabolism and external sources:

$$\begin{array}{cc} q\left(r,t\right) & \left[W\cdot m^{-3}\right] \end{array}$$

If we consider rapid heating of the tissue (i.e., by external MNPs) then the metabolic and convection terms can be neglected. For a multi-layered tissue such as described in Figure 2, we can formulate the problem using this set of PDEs:

(2) $$\left\{ \begin{array}{l} \nabla^{2}\tau_{i}\left(r,t\right)-\begin{array}{cc} \frac{1}{D_{i}}\frac{\partial }{\partial t}\tau_{i}\left(r,t\right)=-\frac{q_{EXT}\left(r,t\right)}{\kappa_{i}}\delta \left[i-N\right] & i=1,2,\ldots ,N \end{array}\\ \begin{array}{ccc} r={\left[\begin{array}{ccc} x & y & z \end{array}\right]}^{T} & x,y,t\in R & z\in \left[z_{i-1},z_{i}\right] \end{array} \end{array}\right.$$

where $\begin{array}{cc} D_{i}=\frac{\kappa_{i}}{\rho_{i}C_{i}} & \left[m^{2}\cdot s^{-1}\right] \end{array}$ is the thermal diffusivity and the subscript *i* is used for denoting tissue properties of the *i*th layer and heating only occurs in the bottom Nth layer. Defining is $z_{i}$ as the cumulative depth: $z_{i}=\sum_{m=0}^{i}d_{m}$, $\tau_{amb}$ and $h_{amb}$ as the ambient temperature and convection coefficient, the set of PDEs is subject to the following boundary conditions:

(3) $$\begin{array}{ll} \underset{x\to \infty }{\lim}\tau_{i}\left(r,t\right)=\underset{y\to \infty }{\lim}\tau_{i}\left(r,t\right)=0 & \text{Temeparture vanishes at inifinte radius}\\ {\kappa_{i}\frac{\partial }{\partial z}\tau_{i}\left(r,t\right)|}_{z=z_{l}}={\kappa_{i+1}\frac{\partial }{\partial z}\tau_{i+1}\left(r,t\right)|}_{z=z_{l}} & \text{The heat flux is continuous over boundaries}\\ {\tau_{i}\left(r,t\right)|}_{z=z_{i}}={\tau_{i+1}\left(r,t\right)|}_{z=z_{i}} & \text{Temperature is continuous over boundaries}\\ -\kappa_{1}\frac{\partial }{\partial z}{\tau_{1}\left(r,t\right)|}_{z=0}=\eta_{up}\left({\tau_{up}-\tau_{1}\left(r,t\right)|}_{z=0}\right) & \text{Heat transfer at upper surface}\\ \kappa_{N}\frac{\partial }{\partial z}{\tau_{N}\left(r,t\right)|}_{z=z_{N}}=\eta_{dn}\left({\tau_{dn}-\tau_{N}\left(r,t\right)|}_{z=z_{N}}\right) & \text{Heat transfer at lower surface} \end{array}$$

To summarize, the problem before us is: given a measurement of ${\tau_{1}\left(r,t\right)|}_{z=0}$ on the skin surface, we wish to estimate $q_{EXT}\left(r,t\right)$ in the Nth tissue layer.

In order to simplify the task at hand, it is suggested to modulate the heat generation in the tissue volume. It can be achieved easily by modulating the high-frequency RF excitation (hundreds of kHz) with a low frequency (several Hz) periodic gating signal:

(4) $$V_{RF}\left(t\right)=A\underset{\begin{array}{l} \text{Fast RF signal to match}\\ \text{the MNP excitation peak} \end{array}}{\underset{︸}{\cos\left(\omega_{RF}t\right)}}\cdot \underset{\begin{array}{c} \text{Slow modulation signal}\\ \text{for resolving the temperature field} \end{array}}{\underset{︸}{\cos\left(\omega_{\mathrm{mod}}t\right)}}$$

Such an excitation will generate heating with the following temporal dependence:

(5) $$q_{Ext}\left(r,t\right)=q_{DC}\left(r\right)+q_{AC}\left(r\right)\cos\left(\omega_{\mathrm{mod}}t\right)$$

Thus the abovementioned PDE set can be reformulated as:

(6) $$\begin{array}{cc} \{\begin{array}{c} \nabla^{2}\tau_{AC,i}\left(r\right)-j\frac{\omega_{\mathrm{mod}}}{D_{i}}\tau_{AC,i}\left(r\right)=-\frac{q_{AC}\left(r\right)}{\kappa_{i}}\delta \left[i-N\right]\\ \nabla^{2}\tau_{DC,i}\left(r\right)=-\frac{q_{DC}\left(r\right)}{\kappa_{i}}\delta \left[i-N\right] \end{array} & i=1,2,\ldots ,N \end{array}$$

Although this presents some simplification of the problem, it is not enough. By taking the spatial Transverse Fourier Transform (spatial-TFT):

(7) $$\begin{array}{l} T_{i}\left(k_{x},k_{y},z\right)={ℱ}_{⊥}\left\{\tau_{i}\left(x,y,z\right)\right\}=\int_{x\in R}\int_{y\in R}\tau_{i}\left(x,y,z\right)e^{j\left(k_{x}x+k_{y}y\right)}dxdy\\ \tau_{i}\left(x,y,z\right)={ℱ}_{⊥}^{-1}\left\{T_{i}\left(k_{x},k_{y},z\right)\right\}=\frac{1}{{\left(2\pi \right)}^{2}}\int_{k_{x}\in R}\int_{k_{y}\in R}T_{i}\left(k_{x},k_{y},z\right)e^{-j\left(k_{x}x+k_{y}y\right)}dk_{x}dk_{y} \end{array}$$

The above PDE set simplifies to the following ODE set:

(8) $$\begin{array}{cc} \{\begin{array}{c} \frac{\partial^{2}}{\partial z^{2}}T_{AC,i}-\left(k_{x}^{2}+k_{y}^{2}+j\frac{\omega_{\mathrm{mod}}}{D_{i}}\right)T_{AC,i}=-\frac{Q_{AC}}{\kappa_{i}}\delta \left[i-N\right]\\ \frac{\partial^{2}}{\partial z^{2}}T_{DC,i}-\left(k_{x}^{2}+k_{y}^{2}\right)T_{DC,i}=-\frac{Q_{DC}}{\kappa_{i}}\delta \left[i-N\right] \end{array} & i=1,2,\ldots ,N \end{array}$$

where $Q\left(k_{x},k_{y},z\right)$ is the TFT of q(x,y,z). These equations have a known homogenous solution:

(9) $$T_{i}^{H}\left(k_{x},k_{y},z\right)=\left\{ \begin{array}{l} \begin{array}{cc} T_{AC,i}=A_{AC,i}e^{\mu_{AC}z}+B_{AC,i}e^{-\mu_{AC}z} & \mu_{AC}=\sqrt{k_{x}^{2}+k_{y}^{2}+j\omega_{\mathrm{mod}}/D_{i}} \end{array}\\ \begin{array}{cc} T_{DC,i}=A_{DC,i}e^{\mu_{DC}z}+B_{DC,i}e^{-\mu_{DC}z} & \mu_{DC}=\sqrt{k_{x}^{2}+k_{y}^{2}} \end{array} \end{array}\right.$$

Thus, the DC equations are just a particular case of the AC equations with $\omega_{\mathrm{mod}}=0$. From this point onward, we won’t separate between the two. If we consider the source term to be of the form $Q\left(k_{x},k_{y},z\right)=\tilde{Q}\left(k_{x},k_{y}\right)\delta \left(z-z_{s}\right)$ then a particular solution suggested by the authors in a previous work is:

(10) $$T_{N}^{P}\left(k_{x},k_{y},z\right)=-\frac{1}{\mu_{N}\kappa_{N}}\tilde{Q}\left(k_{x},k_{y}\right)H\left(z-z_{s}\right)\sinh\left(\mu_{N}\left(z-z_{s}\right)\right)$$

where H(z) is the Heaviside step function. The total solution is:

(11) $$T_{i}\left(k_{x},k_{y},z\right)=T_{i}^{H}\left(k_{x},k_{y},z\right)+T_{N}^{P}\left(k_{x},k_{y},z\right)$$

Now, if we consider a general source distribution:

(12) $$Q\left(k_{x},k_{y},z\right)=\int_{z^{\prime }=z_{N-1}}^{z_{N}}Q\left(k_{x},k_{y},z^{\prime }\right)\delta \left(z^{\prime }-z\right)dz^{\prime }$$

Then the general particular solution will be:

(13) $$T_{N}^{P}\left(k_{x},k_{y},z\right)=-\frac{1}{\mu_{N}\kappa_{N}}\int_{z^{\prime }=z_{N-1}}^{z}Q\left(k_{x},k_{y},z^{\prime }\right)\sinh\left(\mu_{N}\left(z-z^{\prime }\right)\right)dz^{\prime }$$

It should be noted that since in the above expression $z\geq z_{N-1}$, the particular solution is zero in all boundaries, except for $z=z_{N}$. Thus we can consider only the homogeneous solution for all boundary conditions (B.Cs) except for the lower boundary. Applying the continuity B.Cs we get the relations:

(14) $$\begin{array}{l} A_{i}e^{\mu_{i}z_{i}}+B_{i}e^{-\mu_{i}z_{i}}=A_{i+1}e^{\mu_{i+1}z_{i}}+B_{i+1}e^{-\mu_{i+1}z_{i}}\\ A_{i}\kappa_{i}\mu_{i}e^{\mu_{i}z_{i}}-B_{i}\kappa_{i}\mu_{i}e^{-\mu_{i}z_{i}}=A_{i+1}\mu_{i+1}\kappa_{i+1}e^{\mu_{i+1}z_{i}}-B_{i+1}\mu_{i+1}\kappa_{i+1}e^{-\mu_{i+1}z_{i}} \end{array}$$

Or put succinctly into vector-matrix form:

(15) $$\left[\begin{array}{c} A_{i+1}\\ B_{i+1} \end{array}\right]=\underset{M_{i}}{\underset{︸}{\frac{1}{2\mu_{i+1}\kappa_{i+1}}\left[\begin{array}{cc} \left(\mu_{i+1}\kappa_{i+1}+\mu_{i}\kappa_{i}\right)e^{-\left(\mu_{i+1}-\mu_{i}\right)z_{i}} & \left(\mu_{i+1}\kappa_{i+1}-\mu_{i}\kappa_{i}\right)e^{-\left(\mu_{i+1}+\mu_{i}\right)z_{i}}\\ \left(\mu_{i+1}\kappa_{i+1}-\mu_{i}\kappa_{i}\right)e^{\left(\mu_{i+1}+\mu_{i}\right)z_{i}} & \left(\mu_{i+1}\kappa_{i+1}+\mu_{i}\kappa_{i}\right)e^{\left(\mu_{i+1}-\mu_{i}\right)z_{i}} \end{array}\right]}}\left[\begin{array}{c} A_{i}\\ B_{i} \end{array}\right]$$

Denoting the matrix described in Equation (15) by $M_{i}$ one can repeat this process to get the matrix relation (denoted by $M^{tot}$) between the first and last coefficients:

(16) $$\left[\begin{array}{c} A_{N}\\ B_{N} \end{array}\right]=\underset{M^{tot}}{\underset{︸}{M_{N-1}\ldots M_{3}M_{2}M_{1}}}\left[\begin{array}{c} A_{1}\\ B_{1} \end{array}\right]$$

Considering the convection B.C and infinite depth B.C (and accounting for the particular solution there) we get:

(17) $$\begin{array}{l} \begin{array}{cc} A_{1}=\tilde{\alpha }B_{1}+\tilde{\beta }T_{up} & A_{N}=\tilde{\gamma }B_{N}+\tilde{\delta }T_{dn}+T_{Q} \end{array}\\ \begin{array}{cc} \tilde{a}=\frac{\kappa_{1}\mu_{1}+\eta_{up}}{\kappa_{1}\mu_{1}-\eta_{up}} & \tilde{\beta }=-\frac{\eta_{up}}{\kappa_{1}\mu_{1}-\eta_{up}} \end{array}\\ \begin{array}{cc} \tilde{\gamma }=\frac{\kappa_{N}\mu_{N}-\eta_{dn}}{\kappa_{N}\mu_{N}+\eta_{dn}}e^{-2\mu_{N}z_{N}} & \tilde{\delta }=\frac{\eta_{dn}}{\kappa_{N}\mu_{N}+\eta_{dn}}e^{-\mu_{N}z_{N}} \end{array} \end{array}$$

where the source generated temperature $T_{Q}$ is given by:

(18) $$T_{Q}=\frac{1}{2\kappa_{N}\mu_{N}}\int_{z^{\prime }=z_{N-1}}^{z_{N}}Q\left(k_{x},k_{y},z^{\prime }\right)\left[\tilde{\gamma }e^{\mu_{N}z^{\prime }}+e^{-\mu_{N}z^{\prime }}\right]dz^{\prime }$$

Inserting these results into Equation (16) and solving for A_1_, B_1_ yields:

(19) $$\left[\begin{array}{c} A_{1}\\ B_{1} \end{array}\right]=\frac{1}{\left(M_{12}-\tilde{\gamma }M_{22}\right)-\tilde{\alpha }\left(\tilde{\gamma }M_{21}-M_{11}\right)}\left[\begin{array}{cc} M_{12}-\tilde{\gamma }M_{22} & \tilde{\alpha }\\ \tilde{\gamma }M_{21}-M_{11} & 1 \end{array}\right]\left[\begin{array}{c} \tilde{\beta }T_{up}\\ \tilde{\delta }T_{dn}+T_{Q} \end{array}\right]$$

Thus, on the skin surface we get:

(20) $$T_{1}\left(k_{x},k_{y},0\right)=\frac{\left[M_{12}-M_{11}+\tilde{\gamma }\left(M_{21}-M_{22}\right)\right]\tilde{\beta }T_{up}+\left(\tilde{\alpha }+1\right)\tilde{\delta }T_{dn}+\left(\tilde{\alpha }+1\right)T_{Q}}{\left(M_{12}-\tilde{\gamma }M_{22}\right)-\tilde{\alpha }\left(\tilde{\gamma }M_{21}-M_{11}\right)}$$

which is directly related to the source TFT and to the thermal properties of the tissue layer.

By taking the inverse transform we can obtain:

(21) $$\begin{array}{ll} \tau_{1}\left(x,y\right)= & \underset{\tau_{BC}}{\underset{︸}{{ℱ}_{⊥}^{-1}\left\{\frac{\left[M_{12}-M_{11}+\tilde{\gamma }\left(M_{21}-M_{22}\right)\right]\tilde{\beta }T_{up}+\left(\tilde{\alpha }+1\right)\tilde{\delta }T_{dn}}{\left(M_{12}-\tilde{\gamma }M_{22}\right)-\tilde{\alpha }\left(\tilde{\gamma }M_{21}-M_{11}\right)}\right\}}}\\ & +\underset{\tau_{source}}{\underset{︸}{{ℱ}_{⊥}^{-1}\left\{\frac{\tilde{\alpha }+1}{\left(M_{12}-\tilde{\gamma }M_{22}\right)-\tilde{\alpha }\left(\tilde{\gamma }M_{21}-M_{11}\right)}T_{Q}\right\}}} \end{array}$$

Thus, there is a contribution related to the upper and lower B.C and a contribution related to the source. Since $T_{up}$ and $T_{dn}$ are potentially known, one can estimate $q_{DC}\left(r\right)$ or $q_{AC}\left(r\right)$ from $\tau_{1}\left(r\right)-\tau_{BC}\left(r\right)$ by Wiener filtering:

(22) $${\hat{q}}_{est}\left(x,y\right)={ℱ}_{⊥}^{-1}\left(\frac{P^{*}\left(k_{x},k_{y}\right){ℱ}_{⊥}\left\{\tau_{1}\left(x,y\right)-\tau_{BC}\left(r\right)\right\}}{{\left|P\left(k_{x},k_{y}\right)\right|}^{2}{ℱ}_{⊥}\left\{\tau_{1}\left(x,y\right)\right\}+N}\right)$$

where $P\left(k_{x},k_{y}\right)=\frac{\tilde{\alpha }+1}{\left(M_{12}-\tilde{\gamma }M_{22}\right)-\tilde{\alpha }\left(\tilde{\gamma }M_{21}-M_{11}\right)}\frac{1}{2\kappa_{N}\mu_{N}}\left(e^{-\mu_{N}z_{s}}+\tilde{\gamma }e^{\mu_{N}z_{s}}\right)$ is the Thermal Point Spread Function (TPSF) and N is the noise floor of the measurement.

### 2.2. Thermal Phantom and Setup

**Figure 3:**
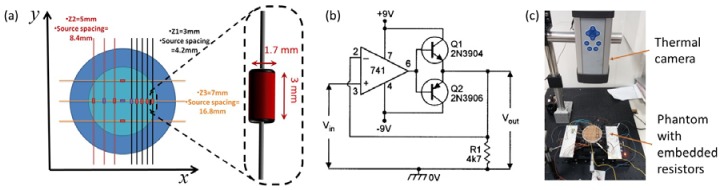
Phantom set-up. (**a**) Sets of resistors embedded in gels with different distances between them horizontally and 3 different depths. The resistors marked with purple are the ones which a thermal couple was embedded with them and was used to verify the actual temperature. All resistors are identical in their thermal and electrical properties. Multiple function generators supply current to the resistors thus creating different controllable temperatures and frequencies; (**b**) An electrical diagram showing the design of the Bidirectional current booster circuit that was used to amplify the function generator’s signal and supply the proper current o the resistors; (**c**) A photo of the actual phantom and thermal camera positioned to capture the entire phantom.

We developed a simple controllable tissue mimicking phantom (Figure 3a) in order to test the model predictions experimentally. The phantom is made from a petri dish (86 mm in diameter and 14 mm in depth) filled with polyester resin (ETERSET3030, Eternal Chemical Co. Ltd., Kaohsiung City, Taiwan) with thermal conductivity and capacity that match those of a human liver tissue [26]. This is shown in Table 1. The thermal properties of agarose gel—a commonly used material for tissue phantoms are listed as well for comparison. It can be seen that the thermal properties of all three are similar. In this research, polyester was chosen over agarose gel due to its stability. Once hardened it stays constant in shape and composition for many months, allowing ample time to conduct multiple and comparable measurements. This is in contrast to agarose gel which loses water quickly thus changing its volume and composition.

The notion behind this phantom was the ability to generate a set of distributed, individually controlled heat sources inside a heat conducting medium and simulate different scenarios with 5 degrees of freedom: 3-dimensional space, time (or modulation frequency) and stimulation power. To accomplish this, the thermal phantom had eleven 120 Ω small electrical resistors (MBA0204 series axial thin film fixed resistor, size 3 × 1.7 mm, Vishay Intertechnology, Inc. Malvern, PA, USA) embedded in it, separated by different spacing between them. The resistors were divided into three groups. The shallowest group at 3 mm depth has five resistors with 4.2 mm spacing; next a 3-resistor group is located at 5 mm depth with 8.4 mm spacing. Finally, at depth of 7 mm, a group of three resistors with 16.8 mm spacing. Resistor groups were far enough from each other to negate any disturbance during imaging. All groups were confined to a central region within the phantom (the cyan zone in Figure 3a) to avoid the effects of the boundaries. This enabled us setting different temperature levels in each of them. Thus, such a phantom simulated a physical scenario of distributed point sources in a radially infinite isotropic, homogeneous medium. While in the axial direction the medium is exposed to thermal natural convection at the top surface and thermally insulated at the bottom.

The resistors energy output, and thus their temperatures are controlled by a multi-output function generator (DS345, Stanford Research Systems, Sunnyvale, CA, USA). Each resistor could be individually controlled electronically in order to simulate temperature variation due to different heat source power. Also, the resistors could be electrically connected in parallel so that the resistors phase and amplitude are matched and the thermal signal will be emitted coherently from a group of resistors. The electrical voltage signal was a zero offset pure sine wave generated by a function generator. Although function generators are attractive for creating various voltage waveforms, they fail to supply the current required for power demanding applications. Thus, the function generator output signal was amplified by a current booster follower circuit as described in Figure 3b in order to supply the sufficient current to the resistors. Since current needs to be both synced and sourced (due to the AC waveform) a bidirectional voltage follower was used. The heat generated by the resistor is proportional to their voltage squared, the thermal response contained both zero frequency DC component as well as an AC component at twice the voltage signal frequency: $q_{Therm}=V^{2}\left(t\right)/R=A^{2}/2R+A^{2}\cos\left(2\omega_{\mathrm{mod}}t\right)/2R$. The amplitude was set to 7.2 V per resistor, which resulted in a 0.432 W of generated heat. However, only half of the heat was attributed to the AC term. This is a fundamental limitation that results from the ability to heat only (without being able to cool the tissue below the nominal temperature). Thus, a DC component will always be present.

A thermal camera (ThermoVision A40M, FLIR Systems, Wilsonville, OR, USA) was positioned at 30 cm above the phantom to capture thermal images for the various applied parameters. The camera has a 320 × 240 pixel array with a built-in lens with a field of view (FOV) of 24° × 18°. Three signals were acquired synchronically: The resistor’s voltage was measured via data acquisition card (USB-6008 National Instruments Austin, Austin, TX, USA) at 10 Hz. The temperature of the close vicinity of some of the resistors (marked in purple in Figure 3a) was measured via a thermocouple at 1 Hz and the surface temperature was measured by a thermal camera at frame rates varying from 30 Hz to 0.3750 Hz while maintaining a factor of 75 over the stimulation frequency. The voltage was applied immediately to the various resistors at the beginning of each experiment, but the data acquisition was halted until all transient effects have vanished. E.g., until the surface temperature (measured by the thermal camera) time dependence resembles a pure sinusoid. In order to achieve high SNR even in relatively high stimulation frequencies, the first set of experiments (one source per depth) was acquired for 24 stimulation cycles. The second set of experiments (multiple sources per depth) was acquired for 12 stimulation cycles. The experimental setup is shown in Figure 3c.

### 2.3. Thermal Image Processing

**Figure 4:**
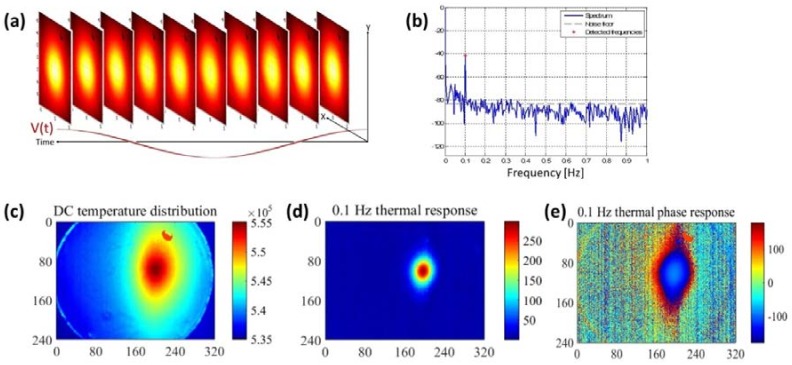
(**a**) A time sequence of thermal images in response to a 0.05Hz sinusoidal voltage; (**b**) The spectrum of a pixel in the image showing the spectral components in DC and 0.1 Hz; (**c**) Magnitude image of the DC component showing the broad response; (**d**) Magnitude image of the 0.1 Hz AC component showing a much narrower response; (**e**) Phase image of the 0.1 Hz AC component that allows easy separation of the signal from the sounding noise.

Surface thermal measurements were processed by taking the temporal Fourier transform and picking only the (complex) image components at $2\omega_{\mathrm{mod}}$ as depicted in Figure 4. The complex images can be represented as the phase and magnitude plots of the temperature. The phase images retain most of the structural/morphological data of the source. The magnitude images indicate the exact transverse position of the source by filtering out spatial information and creating a strong temperature gradient highlighting the maximum value point. For estimating the source distribution, those images were later Weiner filtered, as was described earlier, to estimate the thermal power depth distribution. This process is summarized in Figure 4.

## 3. Results

### 3.1. Validation of the Thermal Model

**Figure 5:**
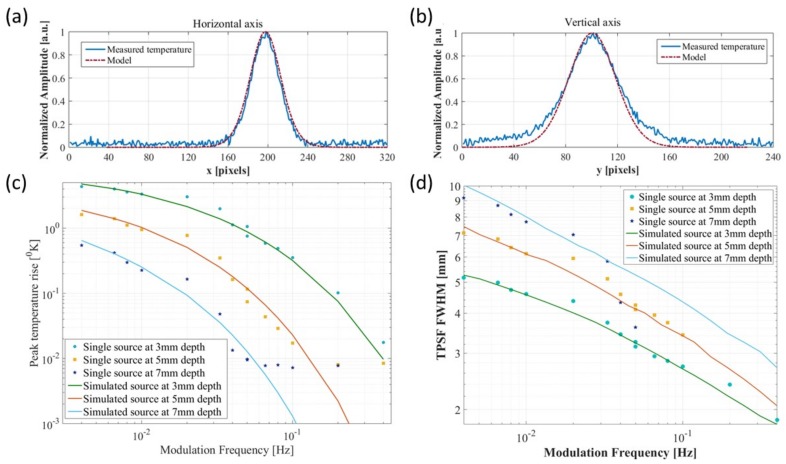
Comparison of the thermal model vs. measurements. (**a**,**b**) Comparison of the predicted PSF with measurement of a single heat source for both Horizontal and vertical axis. These show the high correlation between the theoretical and experimental results; (**c**) Peak surface temperature rise as a function of modulation frequency at different depths; (**d**) Thermal PSF FWHM as a function of modulation frequency at different depths. Those plots show the accuracy of the thermal model is retained at multiple depths and over three orders of magnitude of modulation frequencies.

First, we’ve compared the theoretical predictions with those measured in practice. To that end, only a single resistor was used at each depth and the theoretical temperature profile was calculated. To account for the non-point like source, the calculated Thermal PSF was convolved with a rectangular area of dimensions similar to the resistor in Figure 3 (i.e., 1.7 mm by 3 mm). This simple approximation was sufficient to predict the measured results quite well. The comparison is shown in Figure 5. First, we compared the measurement and the model predicted TPSF at 0.1 Hz. The temperature plots along both the vertical and horizontal axes are shown in Figure 5a,b. One can see that the model predictions are highly correlated with the measurements. The predictions in the horizontal (short) axis are better than those along the vertical (long axis). We suspect that our simple rectangle approximation might be less accurate near the wire connectors. We’ve then moved to compare the Thermal PSF Full Width Half Max (FWHM) and the peak temperature rise on the phantom surface at various depths and modulation frequencies. This is shown in Figure 5c,d respectively. Figure 5c shows that for multiple depths and modulation frequencies, the model predicts the surface temperature rise correctly from 5 °K to 10 °K. At such low level of temperature, the system described in Figure 3 reaches its noise floor and can’t measure the temperature accurately anymore. Figure 5d describes the FWHM as a function of modulation frequency at different depths. Again it is highly correlated with the measured values. The only major deviation is the deepest source at high modulation depths were the SNR is very low. 

### 3.2. Theoretical PSF Analysis

**Figure 6:**
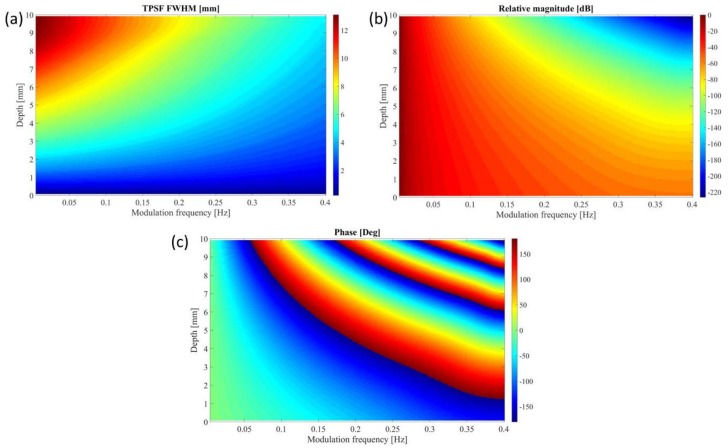
Characterization of the predicted Thermal PSF. (**a**–**c**) The TPSF for different depths and modulation frequencies. (**a**) Shows the FWHM in mm, (**b**) the relative magnitude in dB and (**c**) the phase of the peak.

Encouraged by the high degree of correlation we’ve moved to characterize the predicted thermal PSF as a function of depth and modulation frequency. Figure 6a shows the FWHM of the TPSF. As expected, the deeper the source is or the lower the modulation frequency is, the wider the TPSF. Given a target resolution and a source depth, one can use Figure 6a as a guideline for picking the appropriate modulation frequency. At shallower depths, the resolution degrades slowly with the reduction in frequency. However, in greater depths, the TPSF is less forgiving and the degradation is rapid.

In contrast, Figure 6b shows the relative magnitude of the TPSF. This figure is somewhat a mirror image of Figure 6a. One can see that for low modulation frequencies (up to about 0.1 Hz) the magnitude doesn’t change much with depth. This allows for a high SNR even at great depths (at the expanse of resolution). However, at higher modulation frequencies the magnitude decays very fast with depth which makes such combinations impractical unless a very lengthy measurement is used to increase SNR and compensate for the great loss of signal. 

Finally, Figure 6c presents the phase of the TPSF (at the center). Unlike the amplitude which is dependent on the source strength and the measurement system and thus hard to calibrate, the phase is almost independent of those factors and can be used for estimation of the source depth. As the modulation frequency gets higher the (diffused) Thermal wavelength gets lower and the relation between depth and phase become ambiguous. To negate that, a multi-frequency approach can be used.

### 3.3. Transverse Thermal Resolution Analysis

**Figure 7:**
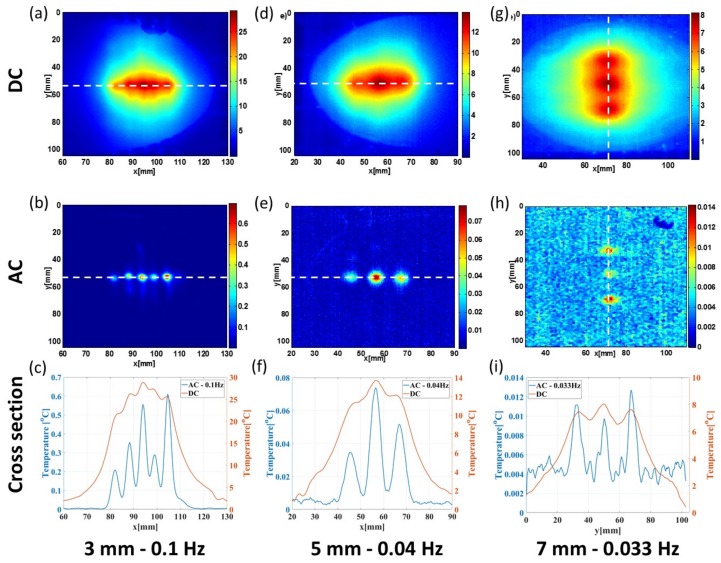
Surface thermal response to an AC stimulated sources at different depths. Presented are the different depths and frequency combinations for the DC and AC components. A cross-section (marked in dashed white line) through both components is also plotted. ((**a**–**c**): 3 mm and 0.1 Hz; (**d**–**f**): 5 mm and 0.04 Hz; (**g**–**i**): 7 mm and 0.033 Hz).

We’ve then moved to estimate the transverse thermal resolution and its improvement due to the modulation. To achieve this we’ve performed simultaneous stimulation of a whole group of resistors at once. Either the five resistors positioned 3 mm beneath the surface with 0.1 Hz modulation frequency (a–c) or the three resistors at a depth of 5 mm with 0.04 Hz modulation frequency (d–f) or the three resistors at depth of 7 mm with 0.033 Hz modulation frequency (g–i). The subfigures of Figure 7 illustrate the magnitude of the surface temperature at DC (top row) and AC (middle row) frequencies. It is clear that the transverse resolution is much improved and allows easy separation between the sources and estimation of their relative intensities. However, the SNR is degrading with the increase in depth and frequency as a result of greater thermal attenuation and thus a reduction in signal amplitude and consequently SNR. One can also appreciate the difference in amplitude. This is due to the difference in resistance (which is also temperature dependent) and the current booster instability.

We define the transverse thermal resolution as the average FWHM of the measured hotspots minus the width of the resistor (1.7 mm). The transverse resolution for each of the measurements presented in Figure 5 is summarized in the following Table 2. It is clear that by modulating the heat source one gets an order of magnitude improvement in the transverse thermal resolution without any further processing. 

### 3.4. SNR Analysis

**Figure 8:**
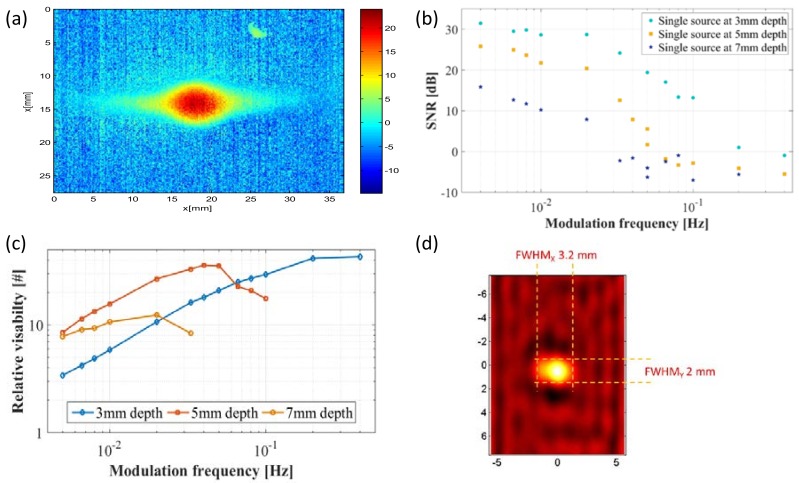
SNR analysis. (**a**) showing an example of the pixel-wise calculate SNR (**b**) Mean SNR in the image as a function of stimulation frequency and source depth; (**c**) Visibility measurements as a function of depth and modulation frequency. The figure shows the relative improvement in visibility compared to DC; (**d**) The reconstruction of a single heat source at 3 mm depth using Weiner deconvolution on the measured modulated surface temperature.

While it is clear that the modulation improves the thermal resolution, it is also clear that the SNR is degraded. We’ve analyzed the SNR in a pixel-wise manner by a built-in Matlab^TM^ “SNR” function. An example of this calculation is shown in Figure 8a. The mean SNR was then calculated for each image. A summary of the mean SNR resulting from a single source as a function of depth and modulation frequency is presented in Figure 8b. It is not surprising that the SNR degrades with depth and with the increase in modulation frequency. Also, the SNR could not be estimated properly below −5 dB due to limitations of the measurement system. At low modulation frequencies up to 0.02 Hz, a slow and gradual decrease in the SNR is shown (which is due to the gradual decrease in the peak temperature rise that was shown in Figure 5c. At higher frequencies, the SNR degrades rapidly with the increase of modulation frequency. This degradation can be somewhat compensated by prolonging the measurement time. As this is a coherent measurement the SNR will increase linearly with the number of cycles used for measurement. 

### 3.5. Thermal Visibility Analysis

The thermal visibility is a measure that combines the effects of improvement in thermal resolution as well as degradation in SNR. In very low modulation frequencies, the TPSF is wide, and each thermal hotspot fills the entire field of view which results in a low contrast. In very high modulation frequencies, the TPSF is narrow but the amplitude is low and disappears into the background noise resulting in low contrast again. In order to find the optimal modulation frequency for each depth, we tested modulation frequencies between 0.005 Hz and 0.4 Hz at all three depths and calculated the hotspot visibility for each scenario. The visibility was defined as the temperature difference divided by temperature average across the line perpendicular to the parallel sources. Figure 6a depicts the improvement in visibility (i.e., *Vis*(*f*,*z*)/*Vis*(0,*z*)) at different depths and modulation frequencies. At shallow 3 mm depths, the modulation can improve hot spot visibility by a factor of up to 43 with a modulation frequency of 0.4 Hz. This advantage degrades with depth and a maximal improvement of 35 at 0.04 Hz at 5 mm and a factor of 12 at a modulation frequency of 0.02 Hz at a depth of 7 mm. Although this limits our ability to see deep into the sample, it is still a considerable improvement over the non-modulated measurements. 

### 3.6. Reconstruction of the Heat Source Distribution

Finally, we applied the Wiener filter for reconstruction of the heat source distribution. This allows us to (partially) negate the effects of both the wide TPSF and decrease in SNR. Moreover it allowed the reconstruction of the heat (rather than temperature) distribution at depth. The reconstruction algorithm was applied to a single source located 3 mm deep with 0.1 Hz modulation frequency. The result is presented in Figure 8d. One can see that the resulting heat distribution only slightly larger than the resistor shape and dimensions (3 mm × 1.7 mm). Moreover, the maximal heat generation seems to be concentrated around the center of the resistor which is more likely than assuming a uniform distribution across its entire footprint.

## 4. Discussion

This paper presents a comprehensive study of a time dependent, externally controlled, distributed heat source for the purpose of focused hyperthermia feedback system. A theoretical rigorous algorithm was developed which predicted a simple relationship between the excitation heat source and the measured surface temperature in the Fourier domain. This relation was exploited to increase the resolution and imaging depth of thermal imaging up to 7 mm deep under tissue surface. Experimental results support this model predictions and highlights its usability for a wide range of applications. Both theoretical as well as experimental analysis was performed to find the best compromise between resolution, depth and SNR. A single visibility was defined and optimized to maximize performances. Following that, a wiener estimation of the heat distribution allowed the solution of the inverse problem thus proving the usefulness of the proposed model-based thermal reconstruction method. 

The results of this research present a method that enables the estimation of deep tissue temperature sources by capturing and processing the transient temperature at the boundary. The proposed method is based on a physical model of the bio-heat transfer equation, then analyses the pre-processed measurement and outputs the temperature field in the vicinity of a heat source. Thereby, allowing the surgeon the ability to dynamically evaluate the hyperthermia treatment efficiency in real time. For example, assess the thermal dose in the cancerous tissue (>10 °C for irreversible damage) and the effect on healthy tissue in its vicinity.

Since the tissue acts as a low-pass filter in the spatial domain, an embedded heat distribution (containing high spatial frequency content) which diffuses to the tissue surface will appear blurred and fuzzy. Therefore, we decided to link the spatial domain to the temporal domain by analyzing the bio-heat transfer model as a function of the modulation frequency under harmonic steady-state condition. Then we showed high a correlation between surface temperature measurements and theoretical model in the space and modulation frequency domain.

Evidently the application of temporal modulation of the heat source power enables the analysis of the surface thermal map in the temporal frequency domain, thus sharpening the image and improving resolution by an order of magnitude (Section 3.3 and Section 3.5). The idea is to separate the measurements to a coherent signal (due to the heat source) and uncorrelated signal, i.e. noise.

Thermal source power and distribution estimation is an ill-posed mathematical inverse problem, hence unstable and very sensitive to measurement and environmental noise. To overcome this obstacle we have utilized the modulated excitation protocol and demonstrated SNR improvement that is proportional to the number of modulation periods. 

Another powerful tool is the theoretical/experimental overview of the TPSF (thermal point spread function). This offers the operator the option to adjust and choose the modulated excitation protocol parameters (modulation frequency) given a set of independent a-priori variables (source depth/distribution and applied power) optimizing a particular scenario and assessing the temperature in the volume of interest. Particular scenario means, different tumors (types and sizes) in variable physiological tissues.

The ideal conditions for temperature reconstruction would be a high-power source unknown distribution in space (compatible for hyperthermia treatment where temperature rise is desired in order to eradicate tumor cells) confined to a known Region of interest (ROI) proximal to tissue surface. As discussed in Section 2.1, there is a significant importance for capturing a large virtual ROI (surface imaged by the thermal camera) in order to fulfill the zero transverse boundary conditions away from the artificial ROI boundaries. Though it may impair the reconstruction resolution (due to camera limited pixel number and size) it will guarantee model compatibility. The results of this study will serve as a strong base for the next step of this research, a nanoparticles based image and treat method and system for solid tumors treatment. The study will be continued on in–vitro models (chicken liver tissue) within 4 months and followed by in vivo studies on small animals bearing tumors. These are all planned to be carried out during 2018.

## Figures and Tables

**Table 1:** Thermal properties of different mediums.

	Thermal Conductivity	Medium Density	Heat Capacitance	Thermal Diffusivity
Polyester Resin	0.155	1090	1670	8.52 × 10^−8^
Agarose Gel 0.75%	0.382	999	4178	9.15 × 10^−8^
Soft Tissue	0.499	1020	3600	1.5 × 10^−7^

**Table 2:** Transverse resolution in different depths.

	Source @ 3 mm	Source @ 5 mm	Source @ 7 mm
Resolution *w*/*o* Modulation	29.12 mm	33.06 mm	58.93 mm
Resolution with Modulation	1.80 mm	3.58 mm	3.89 mm
